# Supplementary material for: A Max-Margin Model for Predicting Residue—Base Contacts in Protein–RNA Interactions
Source: Life (Basel). 2021 Oct 25;11(11):1135. doi: 10.3390/life11111135 (PMC8624843; doi:10.3390/life11111135)
Supplement: Supplementary file 1 [file life-11-01135-s001.zip › life-1427245-supplementary.pdf]

Supplementary material of “A max-margin model for predicting residue-base contacts in protein-RNA interactions”

Shunya Kashiwagi[1], Kengo Sato[1,*], and Yasubumi Sakakibara[1]

[1] Department of Biosciences and Informatics, Keio University, 3–14–1 Hiyoshi, Kohoku-ku, Yokohama 223–8522, Japan

[*]To whom correspondence should be addressed.

S1 Derivation of scoring functions for max-margin training

The loss function of the prediction $\hat{z}$ against the positive data z (Eq. (14) in the main paper) can be transformed into the following using binary-valued variables:

$$
\begin{aligned}
\Delta(z,\hat{z}) =& \delta^{\text{FN residue}}(\text{\# of false negative residues}) \\
&+ \delta^{\text{FP residue}}(\text{\# of false positive residues}) \\
&+ \delta^{\text{FN base}}(\text{\# of false negative bases}) \\
&+ \delta^{\text{FP base}}(\text{\# of false positive bases}) \\
&+ \delta^{\text{FN contact}}(\text{\# of false negative contacts}) \\
&+ \delta^{\text{FP contact}}(\text{\# of false positive contacts}) \\
=& \delta^{\text{FN residue}}\sum_{i=1}^{N_p} I(x_i=1)I(\hat{x}_i=0) + \delta^{\text{FP residue}}\sum_{i=1}^{N_p} I(x_i=0)I(\hat{x}_i=1) \\
&+ \delta^{\text{FN base}}\sum_{j=1}^{N_r} I(y_j=1)I(\hat{y}_j=0) + \delta^{\text{FP base}}\sum_{j=1}^{N_r} I(y_j=0)I(\hat{y}_j=1) \\
&+ \delta^{\text{FN contact}}\sum_{i=1}^{N_p}\sum_{j=1}^{N_r} I(z_{ij}=1)I(\hat{z}_{ij}=0) + \delta^{\text{FP contact}}\sum_{i=1}^{N_p}\sum_{j=1}^{N_r} I(z_{ij}=0)I(\hat{z}_{ij}=1) \\
=& \sum_{i=1}^{N_p}\left\{\delta^{\text{FN residue}}x_i(1-\hat{x}_i) + \delta^{\text{FP residue}}(1-x_i)\hat{x}_i\right\} \\
&+ \sum_{j=1}^{N_r}\left\{\delta^{\text{FN base}}y_j(1-\hat{y}_j) + \delta^{\text{FP base}}(1-y_j)\hat{y}_j\right\} \\
&+ \sum_{i=1}^{N_p}\sum_{j=1}^{N_r}\left\{\delta^{\text{FN contact}}z_{ij}(1-\hat{z}_{ij}) + \delta^{\text{FP contact}}(1-z_{ij})\hat{z}_{ij}\right\}.
\end{aligned}
$$

Here, $I(x_i=1)I(\hat{x}_i=0)=1$ if $\hat{x}_i$ is a false negative and 0 otherwise, and $I(x_i=0)I(\hat{x}_i=1)=1$ if $\hat{x}_i$ is a false positive and 0 otherwise. We also use the fact that $I(x_i=1)=x_i$ and $I(x_i=0)=1-x_i$.

Therefore, the first term of Eq. (13) in the main paper can be simplified into:

$$\begin{aligned}
f_\lambda(P,R,\hat{z})+\Delta(z,\hat{z}) =& \sum_{i=1}^{N_p} u_i\hat{x}_i + \sum_{j=1}^{N_r} v_j\hat{y}_j + \sum_{i=1}^{N_p}\sum_{j=1}^{N_r} w_{ij}\hat{z}_{ij} \\
&+ \sum_{i=1}^{N_p}\left\{\delta^{\text{FN residue}} x_i(1-\hat{x}_i) + \delta^{\text{FP residue}}(1-x_i)\hat{x}_i\right\} \\
&+ \sum_{j=1}^{N_r}\left\{\delta^{\text{FN base}} y_j(1-\hat{y}_j) + \delta^{\text{FP base}}(1-y_j)\hat{y}_j\right\} \\
&+ \sum_{i=1}^{N_p}\sum_{j=1}^{N_r}\left\{\delta^{\text{FN contact}} z_{ij}(1-\hat{z}_{ij}) + \delta^{\text{FP contact}}(1-z_{ij})\hat{z}_{ij}\right\} \\
=& \sum_{i=1}^{N_p}\left\{\left[u_i - \delta^{\text{FN residue}} x_i + \delta^{\text{FP residue}}(1-x_i)\right]\hat{x}_i + \delta^{\text{FN residue}} x_i\right\} \\
&+ \sum_{j=1}^{N_r}\left\{\left[v_j - \delta^{\text{FN base}} y_j + \delta^{\text{FP base}}(1-y_j)\right]\hat{y}_j + \delta^{\text{FN base}} y_j\right\} \\
&+ \sum_{i=1}^{N_p}\sum_{j=1}^{N_r}\left\{\left[w_{ij} - \delta^{\text{FN contact}} z_{ij} + \delta^{\text{FP contact}}(1-z_{ij})\right]\hat{z}_{ij} + \delta^{\text{FN contact}} z_{ij}\right\} \\
=& \sum_{i=1}^{N_p} \bar{u}_i\hat{x}_i + \sum_{j=1}^{N_r} \bar{v}_j\hat{y}_j + \sum_{i=1}^{N_p}\sum_{j=1}^{N_r} \bar{w}_{ij}\hat{z}_{ij} + Const,
\end{aligned}$$

where

$$\begin{aligned}
\bar{u}_i =& u_i - \delta^{\text{FN residue}} x_i + \delta^{\text{FP residue}}(1-x_i) \\
=& \begin{cases} u_i - \delta^{\text{FN residue}} & (\text{if } x_i{=}1) \\ u_i + \delta^{\text{FP residue}} & (\text{if } x_i{=}0) \end{cases} \\
\bar{v}_i =& v_j - \delta^{\text{FN base}} y_j + \delta^{\text{FP base}}(1-y_j) \\
=& \begin{cases} v_i - \delta^{\text{FN base}} & (\text{if } y_j{=}1) \\ v_i + \delta^{\text{FP base}} & (\text{if } y_j{=}0) \end{cases} \\
\bar{w}_{ij} =& w_{ij} - \delta^{\text{FN contact}} z_{ij} + \delta^{\text{FP contact}}(1-z_{ij}) \\
=& \begin{cases} w_{ij} - \delta^{\text{FN contact}} & (\text{if } z_{ij}{=}1) \\ w_{ij} + \delta^{\text{FP contact}} & (\text{if } z_{ij}{=}0) \end{cases} \\
Const =& \sum_{i=1}^{N_p} \delta^{\text{FN residue}} x_i + \sum_{j=1}^{N_r} \delta^{\text{FN base}} y_j + \sum_{i=1}^{N_p}\sum_{j=1}^{N_r} \delta^{\text{FN contact}} z_{ij}
\end{aligned}$$

The last equation indicates that we can maximize the first term of the objective function (13) by replacing scores u_i, v_j and w_{ij} with a constant difference $Const$ that is independent of $\hat{x}_i, \hat{y}_j$ and $\hat{z}_{ij}$.

S2 Hyperparameters

We empirically chose the hyperparameters for the max-margin training: the penalty for positives $\delta^{\text{FN}\,*}$, the penalty for negatives $\delta^{\text{FP}\,*}$, and the weight for ℓ_1 regularization term C. We fixed $\delta^{\text{FP}\,*} = 0.5$ because the balance between $\delta^{\text{FN}\,*}$ and $\delta^{\text{FP}\,*}$ is important. We performed the grid search on $\delta^{\text{FN}\,*} \in \{0.001, 0.005, 0.05, 0.5\}$ and $C \in \{10^n \mid n = -3, \ldots, -6\}$ for the whole dataset. Then, we chose $\delta^{\text{FN}\,*} = 0.05$ and $C = 10^{-5}$ at which good accuracy can be achieved. Tables S1 and S2 show the accuracy under varying $\delta^{\text{FN}\,*}$ and C respectively, indicating that $\delta^{\text{FN}\,*}$ and C are not strongly sensitive to the prediction accuracy.

Supplementary Table

Table S1: Accuracy under varying δ^{FP*} with fixing δ^{FN*} = 0.5 and $C = 10^{-5}$.

	Contacts			Binding residues			Binding bases		
δ^{FP*}	PPV	SEN	F	PPV	SEN	F	PPV	SEN	F
0.001	0.550	0.505	0.513	0.686	0.684	0.670	0.698	0.631	0.647
0.005	0.555	0.504	0.514	0.698	0.684	0.674	0.698	0.630	0.645
0.05	0.539	0.525	0.519	0.664	0.688	0.657	0.684	0.655	0.652
0.5	0.432	0.571	0.476	0.509	0.652	0.554	0.529	0.656	0.570

Table S2: Accuracy under varying C with fixing δ^{FP*} = 0.005 and δ^{FN*} = 0.5.

	Contacts			Binding residues			Binding bases		
C	PPV	SEN	F	PPV	SEN	F	PPV	SEN	F
10^{-6}	0.541	0.519	0.517	0.669	0.677	0.656	0.674	0.646	0.646
10^{-5}	0.539	0.525	0.519	0.664	0.688	0.657	0.684	0.655	0.652
10^{-4}	0.504	0.531	0.501	0.622	0.686	0.631	0.661	0.663	0.644
10^{-3}	0.265	0.392	0.293	0.340	0.509	0.379	0.362	0.498	0.391

Table S3: Structural profiles coding

Residue	α-helix	3-helix	5-helix	folded	β-turn	corner	curl	loop
	H	G	I	E	B	T	S	–
Base	External	Hairpin	Internal	Bulge	Multibranch	Stack		
	E	H	I	B	M	S		

Table S4: Coding of simplified alphabets (10 groups)

amino acids	LVIM	C	A	G	ST	P	FYW	EDNQ	KR	H
coding	L	C	A	G	S	P	F	E	K	H

Table S5: Coding of simplified alphabets (4 groups)

amino acids	LVIMC	AGSTP	FYW	EDNQKRH
coding	L	A	F	E

Table S6: PDB ID and chain IDs used in our test dataset.

PDBID	Protein chain	RNA chain
1AV6	A	B
1C0A	A	B
1DDL	A	D
1DFU	P	M
1DFU	P	N
1DI2	A	C
1DI2	A	D
1DI2	A	E
1F7U	A	B
1FEU	A	B

Table S6: PDB ID and chain IDs used in our test dataset (cont.)

PDBID	Protein chain	RNA chain
1FEU	A	C
1FEU	A	E
1FJG	M	A
1FXL	A	B
1GAX	A	C
1GTF	L	W
1H4S	A	T
1HQ1	A	B
1J1U	A	B
1JBS	A	C
1JID	A	B
1K8W	A	B
1M8W	A	C
1M8W	A	E
1MMS	A	C
1N78	A	C
1OOA	A	C
1Q2R	A	E
1QF6	A	B
1SDS	C	D
1SDS	C	F
1VQ8	1	0
1VQ8	3	0
1VQ8	A	0
1VQ8	B	0
1VQ8	C	0
1VQ8	D	0
1VQ8	D	9
1VQ8	E	0
1VQ8	H	0
1VQ8	H	9
1VQ8	J	0
1VQ8	K	0
1VQ8	L	0
1VQ8	M	0
1VQ8	N	0
1VQ8	N	9
1VQ8	O	0
1VQ8	P	0
1VQ8	Q	0
1VQ8	Q	9
1VQ8	R	0
1VQ8	U	0
1VQ8	V	0
1VQ8	W	0
1VQ8	W	9
1VQ8	X	0
1WPU	A	C
1YZ9	A	C

Table S6: PDB ID and chain IDs used in our test dataset (cont.)

PDBID	Protein chain	RNA chain
1YZ9	A	D
1YZ9	A	E
1YZ9	A	F
1ZH5	A	C
1ZH5	A	D
1ZHO	A	B
1ZHO	A	H
2A8V	A	D
2ANR	A	B
2ASB	A	B
2BGG	A	P
2BGG	A	Q
2BU1	A	R
2E9T	A	B
2E9T	A	C
2FK6	A	R
2FMT	A	C
2GIC	A	R
2J01	I	A
2J01	R	A
2Q66	A	X
2R8S	L	R
2VQE	B	A
2VQE	C	A
2VQE	D	A
2VQE	F	A
2VQE	G	A
2VQE	H	A
2VQE	I	A
2VQE	J	A
2VQE	K	A
2VQE	N	A
2VQE	P	A
2VQE	R	A
2VQE	S	A
2VQE	T	A
2ZUE	A	B
3GIB	A	H
3IEV	A	D
